# A new viewpoint on psychiatry: Supporting children's diversified “ibasho”

**DOI:** 10.1002/pcn5.70361

**Published:** 2026-06-10

**Authors:** Tsubasa Sasaki, Yoshiyuki Hirano

**Affiliations:** ^1^ Research Center for Child Mental Development Chiba University Chiba Japan; ^2^ United Graduate School of Child Development, The University of Osaka, Kanazawa University, Hamamatsu University School of Medicine Chiba University, and University of Fukui Suita Osaka Japan; ^3^ Center for Preventive Medical Sciences Chiba University Chiba Japan

Psychological “ibasho” is one in which one's true self is accepted and where one can have a comforting relationship and a sense of security.^1^ Although the term “ibasho” has traditionally referred to a physical place of belonging in Japanese, it is also used in a psychological sense, as described above. In existing research, various scholars have not only redefined “ibasho” but also explored the relationship between “ibasho” and psychological emotions. In particular, the concept of a “ibasho” is considered to be closely linked to a sense of belonging.^2^ Therefore, in this study, we propose a conceptual definition of “ibasho” as a cultural concept unique to Japan that, in addition to its physical meaning, encompasses a place where individuals can feel psychological comfort and a sense of belonging.

In Japan, it is said that “ibasho” leads to maintaining mental health and forming a cultural identity.^3^ Recent research has shown that a sense of belonging is strongly associated with the mental health and well‐being of children and adolescents. For example, studies have shown that the higher a sense of belonging to school, the lower the incidence of mental health symptoms throughout adolescence.^4^ Furthermore, a sense of belonging has been identified as a key predictor of psychological well‐being.^5^ In other words, feeling “ibasho”—which is associated with a sense of belonging—may be linked to the presence or absence of mental health symptoms. Particularly, “ibasho” is important during childhood, a period of rapid physical and mental development. However, not only is there a lack of sufficient quantitative research on this concept using large‐scale data, but the current state of children's living environments is also changing daily. Examining culture‐specific concepts such as “ibasho” is crucial for deepening our understanding of environmental and relational factors affecting children's mental health in the future. Therefore, this study uses publicly available nationwide data to descriptively identify the distribution and characteristics of children's “ibasho,” while also examining patterns across different life contexts and age groups. By doing so, it provides foundational insights into this cultural concept and proposes perspectives needed for future research exploring children's mental states.

We used publicly available data from a Japanese government survey conducted by the Cabinet Office (2022) (“Kodomo/Wakamono no ishiki to Seikatsu ni Kansuru Chosa”), targeting children aged 10–14 years, to examine the current state of “ibasho.” The data were obtained from e‐Stat, a portal site for Japanese Government Statistics (https://www.e-stat.go.jp/). The total number of survey respondents was 1520. The 1520 participants were asked about their “ibasho” in their rooms, families, schools, online, and within the community. This study focused on the responses provided by these 1520 respondents to the following question:

“Do you feel that the following places are your ‘ibasho’ (i.e., places where you feel comfortable and at ease)?”

Respondents were asked to evaluate multiple locations (room, family, school, internet, and community) using a multi‐point response scale (e.g., agree, somewhat agree, somewhat disagree, disagree, none of the above, and don't know). In this study, we used the proportion of respondents who selected “Agree” or “Somewhat Agree” as an operational definition of “ibasho” (this is also reported as “Agree” in the original dataset). Since the data were in percentage format, actual values were calculated based on the population figures for each aggregated cell. We analyzed trends in perceived “ibasho” by gender and age, as well as correlations across different environments that form these “ibasho.” We also estimated age‐specific trends in perceived “ibasho” through hierarchical clustering.

Figure 1 shows the trends in “ibasho” by age group from the analysis results. While relations between other factors, such as gender, age, and region, and the sense of “ibasho” are documented in the supplementary file. Hierarchical clustering revealed a clear divergence between 10–11‐year‐olds and those aged 12 and above (Figure 1). This suggests that patterns of “ibasho” may change from upper elementary school to junior high school. Existing research reports that during the transition from elementary to junior high school age, children shift from family‐based networks to new social networks like friends and club activities in Japan.^6^ However, this study found that for ages 10–11 and 12 and above, the sense of “ibasho” to places like one's room or the internet increased with age, while the sense of “ibasho” to places in society or school tended to decrease (Figures S1 and S2). Regarding this difference, while the data collection period is the fiscal year 2022, which suggests possible influences from COVID‐19 or social trends, it is difficult to draw conclusions based solely on this data. Nevertheless, it is clear that children's “ibasho” is changing year by year. Overall, this study analyzed the current state of “ibasho” among Japanese children and students aged 10–15 using publicly available data. The findings suggest that as students transition from elementary to junior high school, their perception of “ibasho” shifts from social contexts—such as school and the local community—to more individualized or private contexts, such as their own rooms or the internet. Furthermore, while much of the previous research on “ibasho” has often been limited to conceptual discussions or survey studies, the significance of this study lies in the fact that it quantitatively examined “ibasho” across multiple life contexts by reanalyzing publicly available data and constructing an operational definition of “ibasho.”

**Figure 1:**
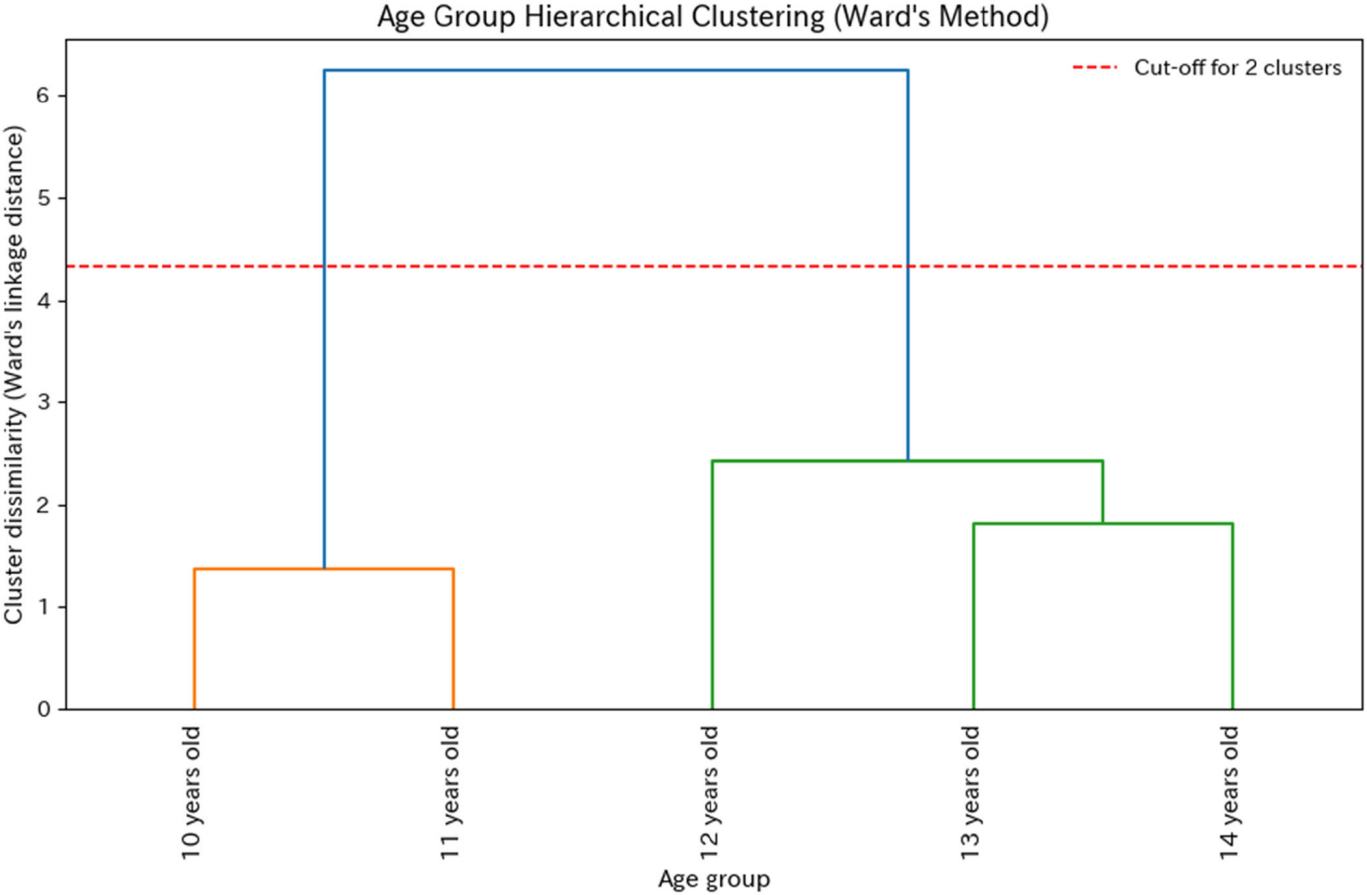
Age group hierarchical clustering. This figure shows the results of hierarchical clustering by age group based on response patterns related to “ibasho,” using the Ward method. The vertical axis represents the linkage distance, indicating the degree of dissimilarity between clusters. The red dashed line indicates the cutoff value used to define the two clusters. The figure shows clusters for the 10–11‐year‐old and 12–14‐year‐old age groups, suggesting that there may be developmental differences in response patterns regarding “ibasho.”

In 2023, the Child and Families Agency, a government agency in Japan, was established to solve problems around children. In cooperation with non‐profit and other organizations, the Children and Families Agency has proposed policies to support the creation of diversified “ibasho” for children and their families.^7^ The existence of various types of “ibasho” has led to the consideration of diverse support methods that take their presence into account. For example, there has been a social demand to create “ibasho” that meets the actual needs of children, and some local governments are supporting “ibasho,” such as Metaverse (VR chat), as “ibasho.”^8^ However, we believe that to appropriately assess children's mental states and conduct research, it is essential not only to update perspectives in response to societal changes but also to pursue research that gets to the heart of what constitutes “ibasho.” Particularly, we believe it would be beneficial to investigate the characteristics of “ibasho” that have a positive impact on children's mental well‐being, as well as how these “ibasho” relate to developmental and cognitive processes to determine whether they truly meet the needs of the children in question. Although this study is based on a secondary analysis of publicly available survey data, incorporating longitudinal and interdisciplinary approaches may yield a deeper understanding of how an “ibasho” relates to children's development and mental health. Furthermore, examining the characteristics of supportive environments will likely help identify factors that promote children's healthy development.

The findings of this study reveal that children's sense of “ibasho” shifts from a group‐based to an individual‐based perspective and diversifies with age. Given this reality, future research aiming to understand children's minds should conduct sociological studies on children's sense of “ibasho”—including support methods—globally, alongside foundational research in fields like cognitive neuroscience. This approach will enable us to grasp the essence of children's psychological sense of “ibasho.” Furthermore, based on these studies, we believe that Japan can eventually improve the mental well‐being of children by having the entire society understand and support children's “ibasho” and discuss the impact on children's mental development.

## AUTHOR CONTRIBUTIONS

Tsubasa Sasaki, under the guidance of Yoshiyuki Hirano, conceived and designed this study, conducted the research, acquired and analyzed the data, and drafted the manuscript. All authors contributed to the preparation of the manuscript and approved its content.

## CONFLICT OF INTEREST STATEMENT

The authors declare no conflicts of interest.

## ETHICS APPROVAL STATEMENT

This study does not require ethical review as it is an analysis using publicly available data accessible to anyone.

## PATIENT CONSENT STATEMENT

This study does not apply because it uses publicly available data.

## CLINICAL TRIAL REGISTRATION

This study does not apply because it uses publicly available data.

## Supporting information

Supporting File 1.

## Data Availability

The data that support the findings of this study are available in e‐Stat (Statistics of Japan) at https://www.e-stat.go.jp/. These data were derived from the following resources available in the public domain: “Kodomo/Wakamono no ishiki to Seikatsu ni Kansuru Chosa,” https://www.e-stat.go.jp/stat-search/files?page=1&layout=datalist&toukei=00100120&tstat=000001203620&cycle=0&tclass1=000001203621&tclass2val=0&metadata=1&data=1.
