# Supplementary material for: A new viewpoint on psychiatry: Supporting children's diversified “ibasho”
Source: PCN Rep. 2026 Jun 10;5(2):e70361. doi: 10.1002/pcn5.70361 (PMC13250696; doi:10.1002/pcn5.70361)
Supplement: Supplementary file 1 — Supporting File 1. [file PCN5-5-e70361-s001.docx]

**Supplementary**


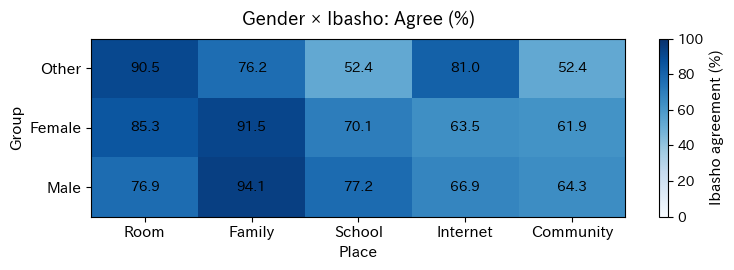


**Supplementary Figure 1. Percentage of respondents who reported feeling a “ibasho”, by gender and place.**

This heat map shows the percentage of respondents who reported feeling a “ibasho” in five different settings home, family, school, the internet, and the local community by gender. The shades of color represent the percentage, with darker colors indicating a higher percentage. “Other” refers to respondents who selected a gender category other than male or female in the original survey.


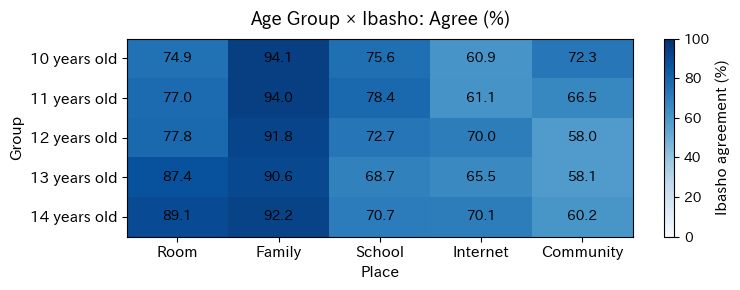


**Supplementary Figure 2. Percentage of respondents who reported feeling a “ibasho” by age group and place.**

This heat map shows the percentage of respondents who reported feeling “ibasho” different settings their room, family, school, the internet, and the local community by age group.

The shades of color represent the percentage, with darker shades indicating a higher percentage.


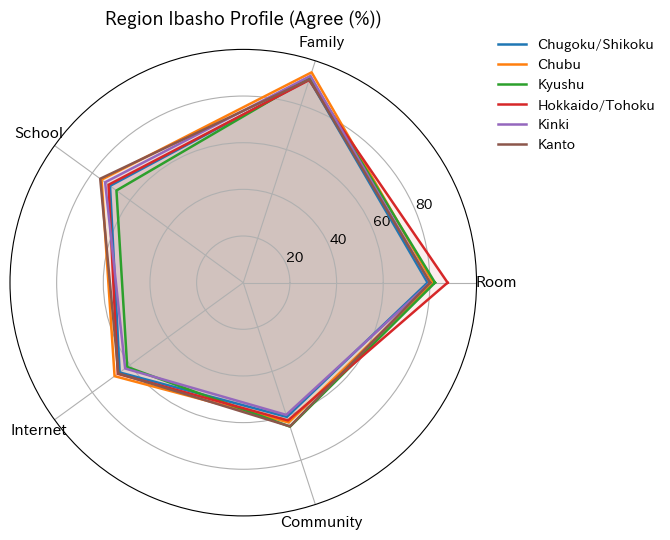


**Supplementary Figure3. Perceptions of “a sense of belonging” by region.**

This radar chart shows the percentage of respondents who reported feeling a “ibasho” in five different settings home, family, school, the internet, and the local community by geographic region. Each line represents a geographic region.
